# Emerging Diluted Ferromagnetism in High‐*T*_c_ Superconductors Driven by Point Defect Clusters

**DOI:** 10.1002/advs.201500295

**Published:** 2016-03-15

**Authors:** Jaume Gazquez, Roger. Guzman, Rohan Mishra, Elena Bartolomé, Juan Salafranca, Cesar Magén, Maria Varela, Mariona Coll, Anna Palau, S. Manuel Valvidares, Pierluigi Gargiani, Eric Pellegrin, Javier. Herrero‐Martin, Stephen J. Pennycook, Sokrates T. Pantelides, Teresa Puig, Xavier Obradors

**Affiliations:** ^1^ Institut de Ciència de Materials de BarcelonaBarcelona08193Spain; ^2^ Department of Physics and AstronomyVanderbilt UniversityNashvilleTN37235USA; ^3^ Materials Science and Technology DivisionOak Ridge National LaboratoryOak RidgeTN37831USA; ^4^ Department of Mechanical Engineering and Materials ScienceWashington University in St. LouisSt. LouisMO63130USA; ^5^ Escola Universitària Salesiana de SarriàBarcelona08017Spain; ^6^ Universidad Complutense de MadridMadrid28040Spain; ^7^ Laboratorio de Microscopías AvanzadasInstituto de Nanociencia de Aragón – ARAIDUniversidad de ZaragozaZaragoza50018Spain; ^8^ ALBA Synchrotron Light SourceCerdanyola del Valles08290Spain; ^9^ Department of Materials Science and EngineeringNational University of SingaporeSingapore117574Singapore

**Keywords:** magnetism, nanostructures, STEM‐EELS, superconductivity, XMCD

## Abstract

Defects in ceramic materials are generally seen as detrimental to their functionality and applicability. Yet, in some complex oxides, defects present an opportunity to enhance some of their properties or even lead to the discovery of exciting physics, particularly in the presence of strong correlations. A paradigmatic case is the high‐temperature superconductor YBa_2_Cu_3_O_7‐δ_ (Y123), in which nanoscale defects play an important role as they can immobilize quantized magnetic flux vortices. Here previously unforeseen point defects buried in Y123 thin films that lead to the formation of ferromagnetic clusters embedded within the superconductor are unveiled. Aberration‐corrected scanning transmission microscopy has been used for exploring, on a single unit‐cell level, the structure and chemistry resulting from these complex point defects, along with density functional theory calculations, for providing new insights about their nature including an unexpected defect‐driven ferromagnetism, and X‐ray magnetic circular dichroism for bearing evidence of Cu magnetic moments that align ferromagnetically even below the superconducting critical temperature to form a dilute system of magnetic clusters associated with the point defects.

## 1 Introduction

Superconductivity and ferromagnetism are considered to be naturally exclusive phenomena, and although they can coexist in high‐quality oxide interfaces between SrTiO_3_ and other nonmagnetic oxides,^1^ and in heavy fermions systems and ruthenocuprates,^2^, ^3^, ^4^ they usually show a competing behavior, like in superconducting/ferromagnetic heterostructures. For instance, in Y123/La_2/3_Ca_1/3_MnO_3_ heterostructures, out‐of‐plane Cu magnetic moments in the superconductor, induced by the Mn moment through interaction across the interface, have been observed.^5^ In fact, layered cuprates also show phase diagrams where the superconducting phase and the antiferromagnetic phase are close to each other because charge, lattice, and spin related phenomena compete, particularly when superconducting and antiferromagnetic ground states are close to each other.^6^ Recent dichroism experiments on undoped and doped cuprates (La_2−_ *_x_*Sr*_x_*CuO_4_, Y123, and Nd_1,2_Ba_1,8_Cu_3_O_7−_ *_x_* thin films and single crystals) have shown the development of field‐induced magnetic moments in both the normal and superconducting states.^7^ The observed dichroic signal, linear with the applied field, was interpreted to arise from action of the Dzyaloshinskii–Moriya (DM) interaction within the CuO_2_ planes, leading to an out‐of‐plane spin canting component, also observed in La_1.85_S_r0.15_CuO_4_/La_0.66_Sr_0.33_MnO_3_ interfaces^8^ (in‐plane antiferromagnetic coupling was also shown by the persistence of collective magnetic excitations observed by resonant inelastic X‐ray scattering).^9^ In addition to that, here we unveil that in Y123 films having a high concentration of Y_1_Ba_2_Cu_4_O_7_ (Y124) intergrowths, complex defects made up of ferromagnetic clusters of Cu and O vacancies are formed, which may act as effective pinning sites at the nanoscale. We also show that the different spatially separated defect clusters display superparamagnetic behavior under high magnetic fields.

High‐temperature superconductor Y123 is indeed among the most relevant playgrounds for the investigation of the physics of magnetic flux lines, in which nanoscale defects are essential to pin vortices and prevent resistive losses in the presence of magnetic fields.^10^ Accordingly, significant efforts have been made in recent years toward nanoengineering Y123 films, such as the assembly of nonsuperconducting phases within the Y123 matrix, which have proven to enhance their performance in power applications.^11^, ^12^, ^13^, ^14^, ^15^, ^16^, ^17^, ^18^, ^19^, ^20^ In chemical solution deposition (CSD) derived Y123 films displaying enhanced vortex pinning efficiency, the introduction of secondary phases within the superconducting matrix renders a huge increase of Y124 intergrowths^19^, ^21^ while keeping critical temperature (*T*_c_) of ≈90 K. The density of intergrowths may vary significantly with the growth method and the processing and in the present study we used CSD derived films in which these intergrowths have higher concentrations.

## 2 Results and Discussion

**Figure 1:**
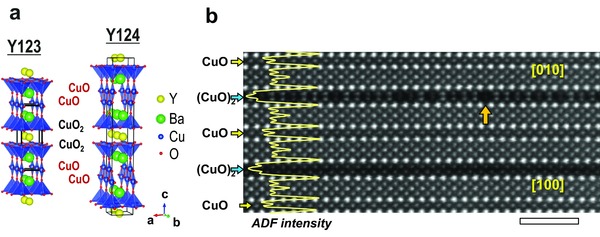
a) Crystal structure of the orthorhombic Y123 and Y124 phases with the repeated sequence of layers. b**)** Atomic resolution image of the Y123 lattice with two Y124 intergrowths imaged along both the [010] and [100] orientation. The Y123 lattice view is easily identified based on its brightest Ba columns, dimmer Y columns in between, and the surrounding least bright square Cu—O lattice. The Cu atoms in the double Cu—O chains have a triangle‐shaped atomic arrangement when viewed along the <100> zone axis, while they lie head‐to‐head when viewed along the <010> zone axis. The vertical arrow points at a pair of Cu vacancies. Scale bar, 2 nm.

The layered structure of Y123 is illustrated in **Figure** **1**a**.** Y124 shares the Y123 structure, but adds one extra Cu—O chain along the Y123 [010] direction or *b*‐axis, with a displacement component of 1/2[0 b c/3]. Since the precursors or targets used for the growth of the films have a stoichiometry of Y123, the extra amount of Cu needed for the formation of Y124 intergrowths would lead to a local Cu off‐stoichiometry, a situation that worsens in the case of Y123 nanocomposites where the number of Y124 intergrowths multiplies (see ref.^18^ and Section S1, Supporting Information for further details of the nanocomposites' microstructure). This Cu off‐stoichiometry might affect the critical temperature (*T*_c_) of the system; however, this detrimental effect is not observed, being the *T*_c_ ≈ 90 K in these films. The reason is that the system balances any Cu deficiency by forming a complex point defect within the double chain. As an example, Figure 1b shows an annular dark field (ADF) Z‐contrast image with two Y124 intergrowths viewed along two different crystallographic directions. Along the [010] direction, the double Cu chain exhibits an irregular contrast that suggests missing atoms. Along the [100] direction, although the image contrast appears homogeneous to the naked eye, the line trace on the left, which shows the laterally averaged ADF signal, indicates, for both crystal orientations, a decrease of the ADF intensity in the (CuO)_2_ planes (or the Cu—O double chains) as compared to the CuO_2_ planes. This reduced intensity, with an uneven distribution of intensity, can be explained by the presence of Cu di‐vacancies, i.e., two Cu vacancies in these atomic columns (*V*_Cu_) in the double chains. High‐resolution electron energy‐loss (EELS) spectrum imaging (Section S1, Supporting Information) also confirms a reduced Cu content on the defects and the presence of O vacancies accompanying the copper vacancies.

**Figure 2:**
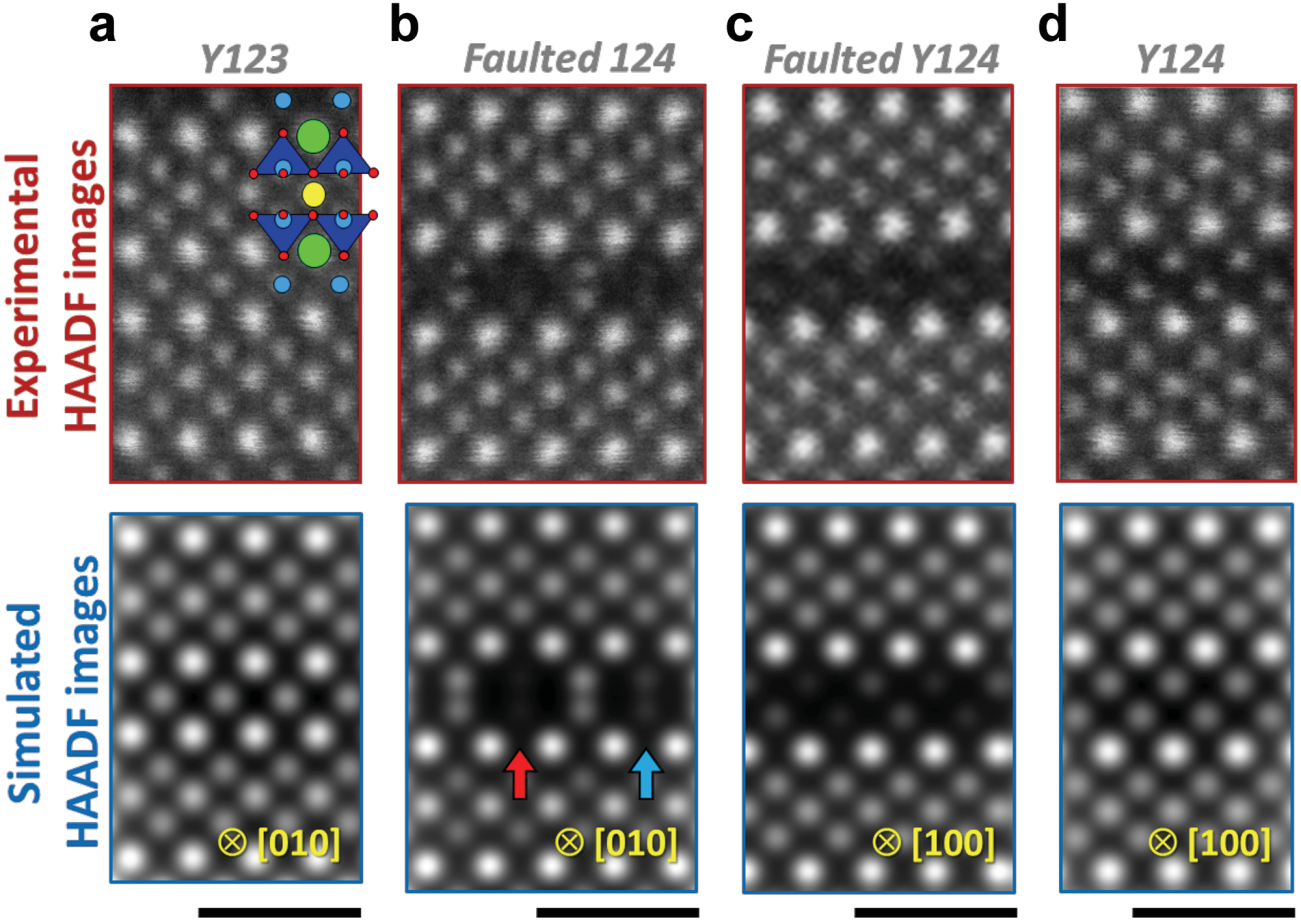
Experimental (upper panel) and simulated (lower panel) Z‐contrast images of Y123, faulted Y124, and fully stoichiometric Y124 phases. a,b) Y123 and faulted Y124 intergrowth images viewed along the [010] zone axis, respectively. c,d) Faulted and fully stoichiometric Y124 intergrowth images viewed along the [100] zone axis, respectively. The simulated image in (b) is performed considering a Cu occupancy of 0.5 and 0.66 in the second (red arrow) and the forth (blue arrow) Cu‐pair columns, respectively. The simulated image in (c) is performed considering a Cu occupancy of 0.5 in two nonconsecutive (010)Y124 planes. For both Y123 and Y124 phases, an approximately 3.5 nm thick supercell was created for the calculations. Scale bars, 1 nm. All Z‐contrast images were acquired from a Y123 + BYTO at 10% nanocomposite thin film.

In order to assess the amount of *V*_Cu_ along a single column of the double Cu—O chain of an intergrowth, images of Y123, faulted Y124, and perfect Y124 structures have been simulated using the STEM_CELL software^22^ (see **Figure** **2**; details of the simulations are given in the Section S1, Supporting Information). Figure 2a shows, as a reference, the experimental and the simulated images of the Y123 structure along the [010]‐zone axis. Simultaneously, faulted Y124 phase images have been simulated along two different zones axes (see Figure 2b,c). All the simulated images are consistent with the experimental observations, and only the one viewed along the *b*‐axis shows clearly the uneven contrast stemming from the presence of the *V*_Cu_. Figure 2d shows the experimental and the simulated images of the fully stoichiometric Y124 structure along the [100]‐zone axis, in clear contrast with that of Figure 2c. In light of these results, we can conclude that the Cu di‐vacancies extend over several unit cells along the *b*‐axis, generating splayed channels with pairs of Cu vacancies along the Y124 intergrowths.

**Figure 3:**
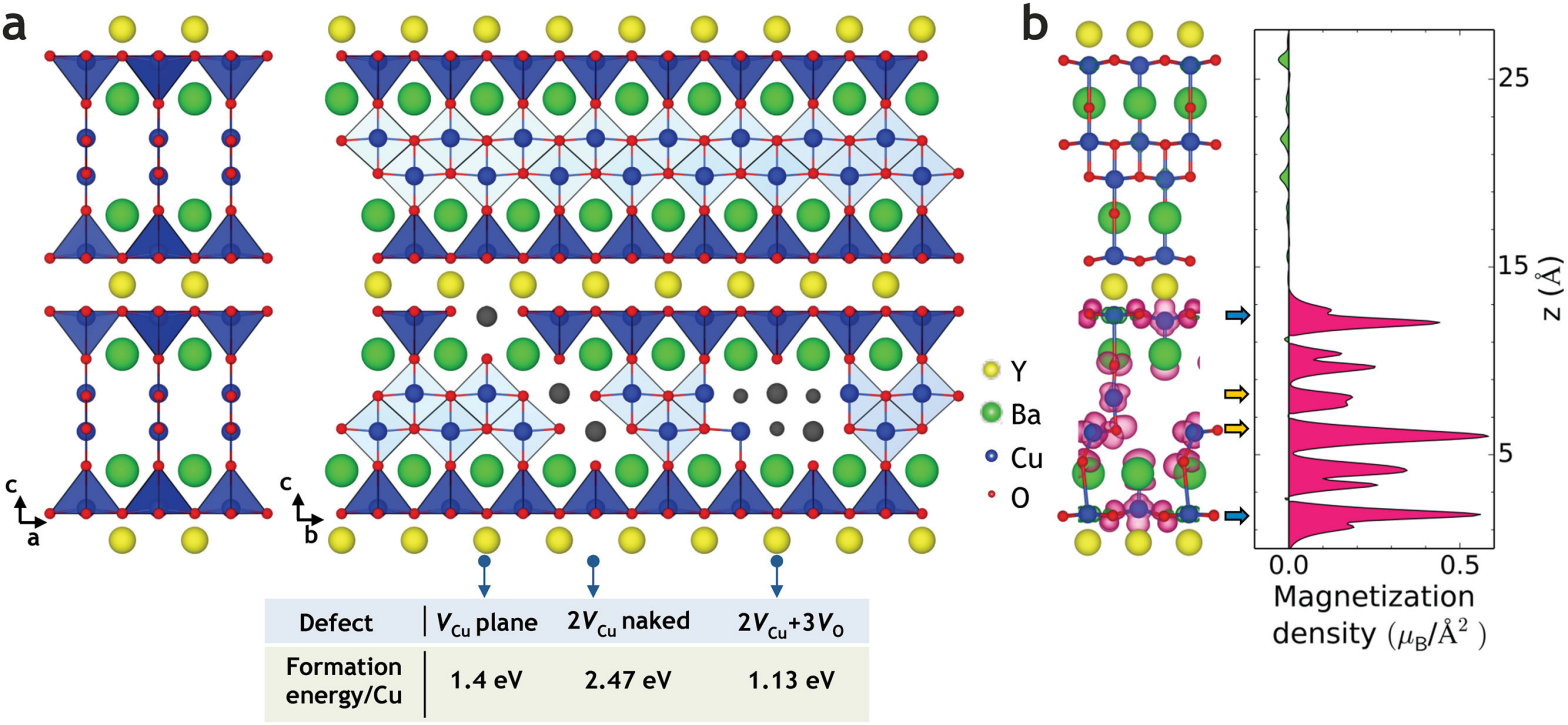
Formation energies and magnetism from DFT calculations. a) Y247 structure and Y247 structure with Cu vacancies at different crystallographic positions and their corresponding formation energies. b) Isosurface plot showing the spin density associated with a 2*V*_Cu_+3*V*_O_ defect in Y248 along with an integrated magnetization/area profile along the *z*‐axis. DFT calculations were performed for a multicell with 60 atoms, comprising 4x YBa_2_Cu_4_O_8_; this implies a multicell with 16 Cu atoms; thus, when a defect of the type 2V_Cu_+3V_O_ appears, there are 14 Cu atoms left. The spin magnetic moment of 2.2 *μ*_B_ per defect cluster is shared by four Cu atoms and their surrounding O atoms. These four Cu atoms have a significant spin moment of 0.325*μ*_B_ each and the remaining 10 Cu have almost no moment.

We have used density functional theory (DFT) calculations to identify the stability of different vacancy configurations by comparing their formation energies.^23^, ^24^ We have used two different supercells, one with an overall stoichiometry of Y_2_Ba_4_Cu_7_O_14_ (Y247), where single and double Cu—O chains alternate, and the other with an overall stoichiometry of YBa_2_Cu_4_O_8_ with only Cu—O double chains. We started by trying Cu vacancies at different crystallographic positions. We find a Cu vacancy at the CuO_2_ plane to be most favorable (*V*_Cu_ plane in **Figure** **3**a) with a formation energy of 1.4 eV. The experimentally observed Cudi‐vacancy in the double chain, as shown in Figure 3a and compatible with microscopy images in Figure 2b, costs ≈1 eV higher per Cu atom than the single Cu vacancy in the plane, and thus seems to be at odds with the microscopy images from the point‐of‐view of defect energetics. Not only does the Cu di‐vacancy have higher formation energy, they also repel each other as a di‐vacancy is found to have a higher formation energy than two separate vacancies in the double chain (2.11 vs 2.47 eV).

A more systematic study taking into account oxygen vacancies in the Cu—O double chains as observed from the O‐elemental mapping (see Section S1, Supporting Information) solves the puzzle. Experimentally, finite concentrations of O vacancies are always present as the films are typically grown at low oxygen partial pressures corresponding to lower oxygen chemical potential. We performed a systematic DFT study of defects comprising different numbers and combinations of Cu and O vacancies (see Section S2, Supporting Information). The results indicate that O vacancies are more favorable in the Cu—Ochains than in the CuO_2_ planes. We find that Cu di‐vacancies decorated by three O vacancies (2*V*_Cu_+3*V*_O_), as shown in Figure 3a, are the most stable type of Cu defect (1.13 eV per Cu vacancy). These results indicate that oxygen vacancies are crucial to stabilize these defect clusters.

We further theoretically investigated the effect of these defects on the electronic and magnetic properties of the system in the metallic phase. An analysis of the DFT electronic structure indicates that the charge density and the occupation of the two Cu *e_g_* orbitals of the ions occupying the CuO_2_ planes differs by <0.1 e/Cu,^25^ with and without the defect. These defect clusters also lead to significant changes in the magnetic properties of the material. While the Y124 phase has no net magnetization, the O‐decorated Cu vacancies give rise to a finite magnetic moment of ≈2.2 *μ*_B_ per 2*V*_Cu_+3*V*_O_ defect cluster. The four Cu atoms neighboring the defect carry a total moment of 1.3 *μ*_B_ (or ≈0.325 *μ*_B_/Cu). Moreover, the hybridization between the 3*d* states of the Cu atoms and the 2*p* states of the intervening O atoms results in a significant magnetic moment of ≈0.9 *μ*_B_ shared by the O atoms neighboring the defect. Figure 3b shows an isosurface plot of the spin density around one such defect cluster, along with a magnetization profile along the *c*‐axis. The magnetic moments of the Cu atoms in the double chain and in the CuO_2_ planes in the vicinity of the defects are of the same sign, i.e., the four Cu moments associated with the 2*V*_Cu_+3*V*_O_ defect cluster are ferromagnetically coupled, as shown in Figure 3b. This magnetic configuration seems to be robust and it is stabilized in the calculations for different initial magnetic configurations. It is important to remark that the magnetization profile extends to the neighboring CuO_2_ planes. The defect structure therefore would be a novel source of multifunctionality and it might also provide an extra vortex pinning contribution, since the magnetic vortices formed by flux lines should find it favorable to go through a nonsuperconducting and magnetic defect with nanoscale dimensions.

Given the high density of these complex defects, in particular within the YBCO nanocomposites, a question that arises is whether the magnetic moments associated with these defects may interact with each other. To tackle this question, we have studied the magnetic coupling between a pair of defect clusters in two double chains in a Y248 supercell by DFT, separated from each other by CuO_2_ planes (≈12 Å) along the *c*‐axis. We find that the induced magnetic moments around the defects in both chains align ferromagnetically, with an energy gain of 10 and 300 meV over the antiferromagnetic and nonmagnetic configurations, respectively. This ferromagnetic coupling between the localized moments associated with the O‐decorated Cu vacancies is reminiscent of dilute‐magnetic semiconductors.^26^, ^27^ However, given the restriction on the size of the supercells that can be used in DFT, it is not possible to comment on long‐range magnetic ordering, especially in a scenario where the 2*V*_Cu_+3*V*_O_ defect clusters are scattered within the Y123 matrix as observed experimentally, and thus not present as an extended network of neighboring vacancy clusters.

**Figure 4:**
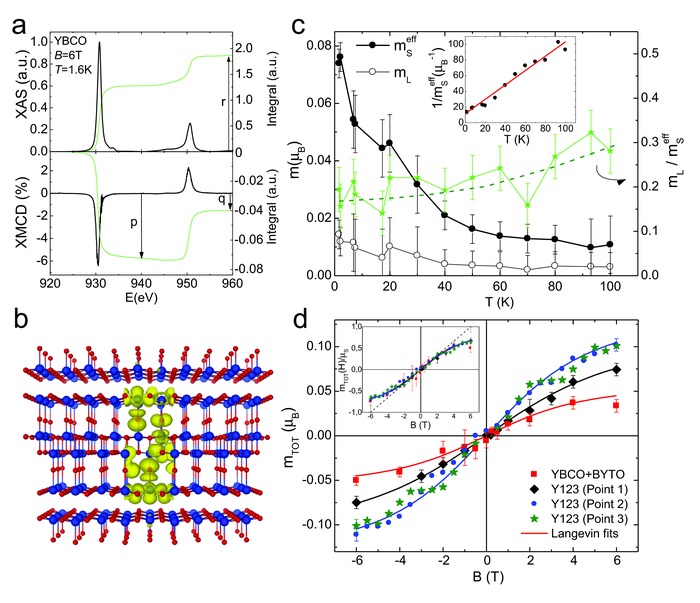
XMCD as a function of the temperature and magnetic field. a) Cu L_2,3_ edge (top) background‐subtracted XAS and (bottom) XMCD spectra measured at 6 T, 1.6 K in normal incidence (*θ* = 0°) for the standard YBCO thin film. The arrows indicate the values of the *p*, *q,* and *r* integrals (right‐hand scales) used for the application of the sum rules. b) 3D model of the magnetic defect cluster, displaying, in yellow, the spin density isosurface of the Y247 supercell. Only Cu and O ions are shown, as blue and red spheres, respectively, for the sake of clarity. c) Temperature dependence of the orbital and effective spin moments and the ratio *m*_L_/*m*_S_^eff^ from 1.6 to 100 K obtained from XAS and XMCD data at 6 T; data acquired at *θ* = 0° after zero‐field cooling (ZFC) the YBCO standard thin film. Inset: inverse of the effective spin moment as a function of the temperature. d) Magnetic field dependence of the total moment (per average Cu in the sample), *m*_TOT_=*m*_S_+*m*_L_, at 1.6 K, *θ* = 0° for the YBCO nanocomposite and the standard YBCO sample at three different points of the sample. The lines are fit to Langevin function Equation (1), with *μ*_c_=1.18 *μ*_B_, *μ*_s_ = 0.06(7) *μ*_B_ for YBCO+BYTO at 10%, *μ*_s_=0.11(0) *μ*_B_ for Y123(1) and* μ*_s_= 0.15(6) *μ*_B_ for Y123 (points 2,3). Different saturation moments *μ*_s_ signal to a variation in the fraction of Cu atoms participating in clusters in each case, as exposed by STEM images, which show a spatial and sample‐to‐sample variation of defect density. Inset: curves normalized to the same *μ*_s_ value.

We have used X‐ray magnetic circular dichroism (XMCD) to experimentally probe the existence of the predicted magnetic moment associated with 2*V*_Cu_+3*V*_O_ defects in YBCO thin films, and to shed light on their possible ferromagnetic interaction. We remind here that XMCD measurements imply recording the difference between the X‐ray absorption spectra (XAS) for left (*I* ^−^) and right (*I* ^+^) circularly polarized photons, and provide element‐specific information on the orbital, spin, and magnetic dipolar moments. XMCD measurements at the Cu *L*_2,3_ edge as a function of the magnetic field, temperature, and beam incident angle were performed in total electron yield (TEY) detection mode on two samples, a pristine Y123 film and a Y123+BYTO nanocomposite thin film, both showing a high Y124 intergrowth concentration within the superficial TEY probing depth (≈6 nm) (see Section S1, Supporting Information). Both samples displayed qualitatively similar Cu *L*_2,3_ edge fine structure and dichroic spectra. The pristine Y123 sample exhibits a clear XMCD spectra for photon energies across Cu L_2,3_ absorption edges, as shown in **Figure** **4**a, which was measured at low temperature (1.6 K) under a 6T field and for X‐ray normal incidence. Measured XMCD is very robust, as large as ≈6.3% at the *L*_3_ white line and with reversed sign at *L*_3_ and *L*_2_ energies. Because XMCD in TEY probes magnetization along the beam direction, this evidences Cu moments at the 3*d* valence band projected along the *c*‐axis, perpendicular to the CuO_2_ planes. Variations in the dichroic signal from 3% to 7.6% within one sample were observed. The percentage of Cu‐XMCD signal measured in our samples is nonlinear with the magnetic field and about a factor of three larger than that reported for sputtered Y123 thin film,^8^ and provides an experimental indication that our proposed defect‐mediated mechanism is active and large in samples with a large Y124 defect density while they lead to a different phenomenology, i.e., diluted ferromagnetism.

The temperature dependence of the Cu moment between 1.6 and 100 K was studied at the maximum field of 6T for the Y123 film. Sum rules^28^, ^29^, ^30^ have been applied to evaluate the Cu orbital moment (*m*_L_) and the effective spin moment (*m*_s_^eff^), assuming the number of holes in the 3*d* shell to be *n*_h_ =1 for Cu^2+^ sites (Section S3, Supporting Information). Figure 4c shows that *m*_s_^eff^(*T*) decreases with the temperature, and as shown in the inset, it follows a Curie dependence with the inverse of the effective spin moment with *T*, which indicates that the system behaves like a superparamagnet.

To gain insight into the origin of the observed magnetic dichroism of the defect clusters, XMCD spectra as a function of the magnetic field in the range between 6 and −6 T were measured in normal incidence at 1.6 K, allowing us to obtain the *m*_s_^eff^(*B*) and *m*_L_(*B*) field dependencies for the two studied samples (Figure S7a, Supporting Information). It should be reminded that for Cu^2+^ sites with high anisotropy, the isotropic spin *m*_s_ moment does not coincide with the effective spin, *m*_s_^eff^(*θ*)=*m*_S_ − 7*m_T_*(*θ*), because the intra‐atomic dipole moment (*m_T_*) is nonnegligible.^31^, ^32^ From XMCD measurements performed in grazing incidence at the so‐called “magic angle” (*θ* = 54.7°) such that *m_T_* cancels, and using sum rules corrected from anisotropy effects,^33^ we determined the isotropic spin moment to be *m*_S_ = 0.064 ± 0.005 *μ*_B_ at 6 T and 1.6 K (Section S3, Supporting Information); hence, we estimate that *m*_S_ amounts ≈80% of $m_{s}^{\mathrm{eff}}$ in normal incidence. Besides, the orbital moment is nonnegligible, contributing about ≈20% to the total moment, *m*_TOT_ = *m*_S_ + *m*_L_ ≈ 0.078 *μ*_B_, at 6 T and 1.6 K.

In order to obtain the Cu total moment field‐dependence $m_{\mathrm{TOT}}(B)$ plots with more data points, and study the Cu total moment field‐dependence spatial variation within one sample, XMCD(B) measurements between −6 and +6 T at different points of the standard YBCO sample were performed by following the resonant *L*_3_ peak intensity as a function of the field. The absolute XMCD scale was fixed at *B* = 6T to the value of *m*_TOT_ derived from a full energy range XMCD (6T) scan. The resulting $m_{\mathrm{TOT}}(B)$ curves for all the studied samples at different points are summarized in Figure 4d, being the $m_{\mathrm{TOT}}(B)=0.8m_{s}^{\mathrm{eff}}(B)+m_{L}(B)$. As shown in the inset, the Cu total moment field dependence clearly deviates from linearity and tends to saturate at high fields, and there is no hysteresis, neither remanence at zero magnetic field. Remarkably, the field dependence of the total magnetization can be well fitted, within a model of superparamagnetic clusters including ferromagnetically ordered moments,^34^ by the Langevin function, $L(\mu_{c})$:

(1) $$m_{\mathrm{TOT}}^{\exp}\left(B\right)\approx \mu_{s}•�\left(\mu_{c}\right)=\mu_{s}•\left[coth\left(\frac{\mu_{c}B}{k_{B}T}\right)-\left(\frac{k_{B}T}{\mu_{c}B}\right)\right]$$

where the saturation value *μ*_S_ corresponds to the total magnetic moment per average Cu atom in the sample, while *μ*_C_ (related to Langevin function curvature) gives the magnetic moment of each individual cluster. The fitting parameters are summarized in the caption to Figure 4d, while a schematics of the defect cluster is shown in Figure 4b. Differences in m_s_ values between the pristine Y123 sample and the nanocomposite are similar to variations within one sample, and are attributed to different local concentrations of point defects. However, note that all the curves collapse when normalized by the saturation value (Figure 4d, inset), indicating that the moment associated with a defect cluster is approximately the same in every case, *μ*_c_ = 1.18 *μ*_B_ ± 0.20 *μ*_B_.

Let us examine these results under the light of the structural information and DFT calculations presented above. As discussed earlier (see Figure 3b), the four Cu atoms neighboring the defect carry a spin‐only moment (*m_s_*) of 1.3*μ*_B_ per defect (the total moment would be ≈20% higher considering the spin‐orbit coupling), which is in excellent agreement with the experimental cluster moment *μ*_c_ determined by XMCD at the Cu L_3,2_ edge. Therefore, combined together, the DFT calculations and XMCD experiments provide strong evidence on the presence of “magnetic cluster” due to the complex 2V_Cu_+3V_O_ defect. Moreover, the DFT predicted saturation moment, equal to the cluster moment divided by the total number of magnetic and nonmagnetic Cu atoms in the cell, 1.56*μ*_B_/14 = 0.11*μ*_B_/Cu, is in the order of the experimental *μ*_s_, implying that a major a fraction (*f* ≈ 1) of Cu atoms participate in clusters (Section S3 of Supporting Information for more details). Although estimation of the number of defects present in the samples is extremely difficult, in view of the scanning transmission microscopy (STEM) cross‐section images (see Section S1 of Supporting Information), one could consider an extreme scenario in which the top‐most part of the samples (5 nm from the surface) consists of pure Y124, which is not unreasonable in view of the scanning transmission mcroscopy (STEM) cross‐section images (see Section S1 of Supporitng Information). Considering the fact that twice as much Cu in the Cu—O chains is needed to form the Y124 out of the Y123 phase, one can conclude that every other unit cell contains two Cu vacancies, just as the structure predicted by DFT. Therefore, it is concluded that *f* ∼1 of the sample sensed by TEY contains faulted cells.

## 3 Conclusions

We have unveiled the complexity of the so‐called Y124 intergrowths, the most common defect in Y123 thin films. Using a combination of STEM imaging, EELS, and DFT calculations, we propose that these common intergrowths should be pictured as double chains with quasi‐1D arrays of defect clusters including two Cu vacancies decorated by three O vacancies embedded in particular chains. Using DFT calculations, we predicted unexpected magnetic moments associated with these defect clusters, with ferromagnetic intercluster interactions between the Cu and O ions, which we have confirmed using XMCD experiments. A model is proposed in which the magnetism of Cu atoms in superconducting Y123 films is explained in terms of a superparamagnetic behavior of isolated ferromagnetic clusters, even below the superconducting transition temperature. A novel dilute ferromagnetic‐superconducting multifunctionality is therefore demonstrated, as the ferromagnetic order of those Cu belonging to the CuO_2_ planes and surrounding the O‐decorated Cu vacancies does not vanish when superconductivity sets in at *T*_c_. Instead, their ferromagnetic state concurs/coexists with the superconducting state of the rest of Cu of the superconducting planes. The presence of these two phases, and the shortness of the superconducting coherence length of Y123, may lift the degeneracy of the spin‐up and spin‐down of the Cooper pairs and cause a pair breaking. Owing to the fact that the density of the Y124 intergrowths is strongly increased in nanocomposite cuprate films, in parallel to a huge enhancement of vortex pinning efficiency,^19^ one may wonder if the novel ferromagnetic behavior could play a role in pinning vortices. In addition, many superconducting materials have an anisotropic structure similar to YBCO and could therefore have similar complex point defects, opening up the possibility to search for the presence of magnetism in the vicinity of superconductivity in all those candidate materials. Our results may also be generalized to other defective perovskite‐based systems in which correlation effects between defect sites might bring new functionalities, as shown by a recent theoretical work on oxygen‐deficient SrTiO_3_, which predicts that different configuration of clustered oxygen vacancies may yield various degrees of magnetization as a result of the interaction between localized magnetic moments at the defect sites.^35^

## 4 Experimental Section

*Samples' Growth* **:** Y123 thin films and nanocomposites were grown by Chemical Solution Deposition. Pristine Y123 thin films were prepared from a methanol‐based metal‐organic solution containing stoichiometric amounts of Y, Ba, and Cu anhydrous trifluoroacetates with a cation molar ratio Y:Ba:Cu of 1:2:3.^36^ This solution was spin coated on 5 × 5 mm LaAlO_3_ (LAO) single crystal substrates and subsequently exposed to a pyrolysis and crystallization steps to obtain epitaxial *c*‐axis oriented films. The crystallization step takes place at 810–830 °C in a wet atmosphere of 0.2 mbar of oxygen (P_O2_) and the oxygenation process is performed at 450 °C and 1 bar of oxygen pressure. Further details on film preparation can be found elsewhere.^37^ Nanocomposites Y123‐MO (MO= BaZrO_3_, Ba_2_YTaO_6_) thin films were prepared by adding molar percentages (0–20% m) of Zr acetate or Ta ethoxide to the original Y‐Ba‐Cu metal‐organic precursor solution. Ba and/or Y anhydrous trifluoroacetate salts have been also added to the precursor solution to preserve the desired stoichiometry Y:Ba:Cu = 1:2:3. Film deposition and processing were performed under the same conditions of the pristine films, described above.^38^ Y_2_Ba_4_Cu_8_O_16_ (Y124) thin films have been prepared from spin coating an anhydrous metal‐organic solution of Y, Ba, Cu trifluoroacetate salts with a cation molar ratio Y:Ba:Cu of 1:2:4 on LAO single crystals. The pyrolysis process was carried out following the Y123 procedure^37^ and the crystallization step takes place at 770–820 °C and 1 bar of oxygen pressure.^39^, ^40^, ^41^

*STEM and EELS Characterization*: For the electron microscopy analyses, we used aberration corrected microscopes, which allow for obtaining atomic resolution images (sub‐Ångstrom resolution). We combined several techniques. On one hand we used high angular annular dark field (HAADF) imaging mode, in which the contrast results from the high angle scattering strength. The intensity in the micrographs is approximately proportional to Z^2^, giving rise to so called Z‐contrast imaging.^42^, ^43^, ^44^, ^45^

We also used STEM in combination with EELS to study the chemistry of the defects found in this work, which permits simultaneous real space studies of structure and chemistry. Aberration correction in the STEM allows for increased contrast and resolution both in imaging and spectroscopy.^46^, ^47^

High‐resolution HAADF STEM studies were carried out on a FEI Titan (60–300 kV) equipped with a probe‐aberration corrector, a monochromator, and an XFEG electron gun. In the HAADF Z‐contrast imaging a probe convergence angle of 25 mrad and an annular dark‐field detector with an inner angle greater than 60 mrad were used. High‐resolution HAADF STEM and EELS data were also acquired in a dedicated STEM, a Nion UltraSTEM, operated at 200 kV and equipped with a fifth‐order Nion aberration corrector. In this case, for the HAADF Z‐contrast imaging a probe convergence angle of 30 mrad and an annular dark‐field detector with an inner angle greater than 86 mrad were used. For EELS, the collection semi‐angle was 48 mrad. The principal component analysis (PCA) method was used to eliminate noise in atomic‐resolution EELS elemental maps.^48^ EELS elemental maps were generated by integrating the PCA‐treated spectra over a 40 eV window, after background subtraction. The acquisition time was 0.03 s per pixel. The HAADF–STEM simulations were performed using the STEM_CELL software. Specimens for STEM and conventional TEM were prepared by conventional methods, by grinding, dimpling, and Ar ion milling.

*XAS and XMCD Measurements*: Soft X‐ray absorption and magnetic dichroism experiments at the Cu L_2,3_ edge were carried out in TEY detection mode at the BL29 BOREAS beamline at the ALBA synchrotron radiation facility, provided with a high‐field vector magnet (HECTOR) end station. The magnetic field, applied in the direction of the beam, could be swept from +6 to −6T at a rate of 2T min^−1^, and the temperature could be decreased to 1.6 T. The sample surface (*ab*‐plane) is mounted in the vertical plane, and measurements were performed in normal incidence, with the *c*‐axis parallel to the beam (*θ* = 0°) as well as in grazing incidence. To rule out experimental artifacts and reduce drift phenomena, XMCD was measured either changing the light helicity or the field direction. The XMCD(*B*) spectra were determined from up to 8 Cu L_2,3_ XAS spectra with right‐ and left‐handed circular polarizations.

*DFT Calculations*: DFT calculations were performed using the Vienna ab‐initio Simulation Package.^49^, ^50^ The influence of the core electrons was incorporated using projector augmented wave potentials^51^ within the spin‐polarized Perdew–Burke–Ernzerhof 52 exchange‐correlation functional. The plane‐wave cutoff energy was set at 500 eV and the ionic relaxation was continued until the forces were smaller than 1 meV/Å. The defects were simulated in two different supercells, one with two double chains along the *z*‐direction and a stoichiometry of YBa_2_Cu_4_O_8_, and the other with one single chain and two double chains along the *z*‐direction and a stoichiometry of Y_3_Ba_6_Cu_11_O_23_. For both the stoichiometries, a $\sqrt{2}\times \sqrt{2}\times 1$ supercell was used, as it was necessary to simulate the di‐vacancies. The Brillouin zone was sampled using a (6×6×1) Monkhorst–Pack *k*‐point mesh^53^ for relaxations and a denser (10×10×2) mesh for the electronic calculations. Some calculations were further repeated in larger (2 × 2 × 1) supercells. The formation energies of the defects obtained using different supercells were found to be within 0.1 eV.

## Supporting information

As a service to our authors and readers, this journal provides supporting information supplied by the authors. Such materials are peer reviewed and may be re‐organized for online delivery, but are not copy‐edited or typeset. Technical support issues arising from supporting information (other than missing files) should be addressed to the authors.

Supplementary
