# Supplementary material for: Emerging Diluted Ferromagnetism in High‐T c Superconductors Driven by Point Defect Clusters
Source: Adv Sci (Weinh). 2016 Mar 15;3(6):1500295. doi: 10.1002/advs.201500295 (PMC5069566; doi:10.1002/advs.201500295)
Supplement: Supplementary file 1 — Supplementary [file ADVS-3-0n-s001.pdf]

Copyright WILEY-VCH Verlag GmbH & Co. KGaA, 69469 Weinheim, Germany, 2016.

ADVANCED SCIENCE
Open Access

Supporting Information

for *Adv. Sci.*, DOI: 10.1002/advs.201500295

Emerging Diluted Ferromagnetism in High-T_c Superconductors Driven by Point Defect Clusters

Jaume Gazquez, Roger. Guzman, Rohan Mishra, Elena Bartolomé, Juan Salafranca, Cesar Magén, Maria Varela, Mariona Coll, Anna Palau, S. Manuel Valvidares, Pierluigi Gargiani, Eric Pellegrin, Javier. Herrero-Martin, Stephen J. Pennycook, Sokrates T. Pantelides, Teresa Puig, and Xavier Obradors*

Emerging diluted ferromagnetism in high-T_c superconductors driven by point defect clusters

J. Gazquez[1,*, †], R. Guzman[1], R. Mishra[2,3,4, †], E. Bartolomé[5], J. Salafranca[6,3], C. Magén[7], M. Varela[6,3], M. Coll[1], A. Palau[1], S. M. Valvidares[8], P. Gargiani[8], E. Pellegrin[8], J. Herrero-Martin[8], S. J. Pennycook[9], S.T. Pantelides[2,3], T. Puig[1], X. Obradors[1]

[1]Institut de Ciència de Materials de Barcelona, Spain

[2]Department of Physics and Astronomy, Vanderbilt University

[3]Materials Science and Technology Division, Oak Ridge National Laboratory

[4]Department of Mechanical Engineering and Materials Science, Washington University in St. Louis, St. Louis, MO 63130, USA

[5]Escola Universitària Salesiana de Sarrià (EUSS), Barcelona, Spain

[6]Universidad Complutense de Madrid. Madrid 28040, Spain

[7]Laboratorio de Microscopías Avanzadas, Instituto de Nanociencia de Aragón – ARAID, Universidad de Zaragoza, Zaragoza 50018, Spain

[8]ALBA Synchrotron Light Source, Barcelona, Spain

[9]Department of Materials Science and Engineering, National University of Singapore

*To whom correspondence should be addressed. E-mail: jgazqueza@gmail.com

[†]These authors contributed equally to this work

This PDF file includes:

S1. Structural analysis

S2. Computational details

S3. XMCD sum rules

Supplementary Figures S1 to S10

Tables S1 and S2

References

S1. Structural analysis

Microstructural analysis. **Figure S1a and S1b** are low magnification HAADF images showing, respectively, the global microstructure of a standard Y123 thin film and a Y123 nanocomposite. These Z-contrast Scanning Transmission Electron Microscopy (STEM) images were acquired with a FEI Titan (60-300 kV) equipped with a probe-aberration corrector. In the HAADF Z-contrast image Y124 intergrowths appear as horizontal black stripes. The Y124 planar defects discussed in the manuscript are common structural defects in both films; numerous intergrowths are observed close to the surface in the two samples. However, the introduction of secondary phase nanoparticles into the Y123 matrix dramatically increases the density of Y124 planar defects in the central bulk part of the nanocomposite thin film.

Figure S2a, **b** and **c** (upper panels) shows high resolution HAADF images of three different films. **a** corresponds to a Y123-BZO nanocomposite film, **b** to a Y123-BYTO nanocomposite films and **c** to a standard Y123 one. In all samples the Y124 intergrowths show the presence of Cu vacancies (see lower panels). It can also be observed that the number of Cu vacancies depends on the concentration of Y124 intergrowths. In areas with a higher concentration of intergrowths, like in the nanocomposite, there is a large number of Cu vacancies (**Fig.S2a**), whereas in other regions, like the central part of the standard film, which present a much lower density of Y124 intergrowths, there are only a few of them (**Fig.S2c**).

EELS elemental mapping. This technique confirmed the presence of Cu vacancies within the double Cu-O chain. **Figure S3a** shows the HAADF image of an isolated Y124 intergrowth decorated with pairs of Cu vacancies. The square marks the region where an EELS spectrum image was recorded. Panels **b-d** of **Figure S3** show the simultaneously acquired ADF signal and the atomic resolution elemental maps of the Ba $M_{4,5}$, O K and Cu $L_{2,3}$ edges, respectively, while **Figure S3f** shows the averaged profile of the Ba M (in green), O K (in red) and Cu L (in blue) signals perpendicular to the double Cu-O plane, along the direction of the white arrow. Both the O K and the Cu L edge signals decrease along the double Cu-O chain stripe (arrow in red). This is a clear indication that the Cu vacancies are accompanied by O vacancies.

Figure S4 shows two high resolution HAADF images of the Y124 phase from two different samples, a Y123 nanocomposite film (**a**) and a Y124 film (**b**). The Y124 phase of the Y123 nanocomposite film exhibits Cu vacancies along the double CuO chains, whereas the Y124 film does not present them. Also, the double CuO chains have different intensity than those in **b**, reflecting different Cu occupancies in different sites along the chains.

HAADF Image simulation. The HAADF–STEM simulations were performed using the STEM_CELL software [1], a simulation package based on Kirkland routines [2]. This software is based on the multislice technique in the 'frozen-lattice' approximation.

Y123 and Y124 supercells, with approximately 3.5nm thickness, were created in order to simulate the contrast modulations due to the presence of Cu vacancies within the double CuO chain (**Figure S5**). Experimental HAADF images in **Figure 2** and **Figure S5** were obtained in a Nion UltraSTEM microscope operated at 200KV. Corresponding simulated images were computed using an aberration corrected probe, with a defocus value of 0nm, a probe forming aperture of 30mrad, C_s=0mm, C_5=50mm, and an acceleration voltage of 200KV. The angle of the ADF detector in the calculations was 80-240mrad. Source size broadening was taken into account using a Gaussian with 0.7Å FWHM.

Two sets of Y124 super-cells were simulated, one with V_{Cu} and another without vacancies, both along two different zone axes, i.e. along [010] and [100] directions, see **Figure S5a** and **b** respectively. To be consistent with the experimental images (shown in **Figure 2**), the concentration of vacancies was varied over a wide range. For the faulted Y124 along the [010] zone axis, the best match between the simulated and the experimental images required a Cu occupancy of 0.5 and 0.66 down the columns along the second (red arrow) and the fourth (blue arrow) Cu-pair columns of the image, respectively. The Cu atoms subtraction was carried out removing non-consecutive Cu-pairs along the b-axis, while the other three Cu-pair columns of the super-cell were kept fully occupied, as shown schematically in the lower panel. For the [100] zone-axis case, **Figure S5b**, a Cu occupancy of 0.5 was necessary to match the contrast of the simulated image with the experimental one. Notice that only the simulated image viewed along the b-axis shows the contrast variation stemming from the presence of the Cu vacancies, being dimmer the Cu-pair column where half of the Cu atoms are missing. Nonetheless, in the [100] case, the simulated image of the faulted Y124 structure shows a general decrease of the HAADF intensity, in agreement with the experimental observations.

S2. Computational details

Defect formation energies and chemical potentials. We use density functional theory (DFT) to determine the most stable Cu vacancies by calculating their formation energies. This requires the knowledge of the chemical potential of individual copper (μ_{Cu}) and oxygen (μ_O) atoms within the compound, which is not well defined. Instead, we vary μ_{Cu} and μ_O over a range of values bound by any two phases of Cu and O that are considered to be under thermodynamic equilibrium[3]. For "O-deficient" (equivalent to "Cu-rich") conditions corresponding to lower oxygen chemical potential, we use the energy of a Cu atom in bulk Cu (with face centered cubic lattice) as μ_{Cu} and derive μ_O from the difference in energies of CuO and Cu. Similarly, for "O-rich" conditions, we use half of the energy of an oxygen molecule in its most stable triplet state as μ_O and determine μ_{Cu} from the difference in energy of CuO and μ_O. Under "O-rich" conditions, where the concentration of oxygen vacancies is extremely small, we find the most favorable site for copper vacancies to be at the superconducting CuO_2 planes (**Figure 3a**), with a formation energy of 0.23 eV per Cu atom. They are significantly more stable than the pair of Cu vacancies in the CuO double chains as observed from the STEM images (see **Figure 1b or 2b** of the main manuscript, for instance) which have a formation energy of 1.4 eV per Cu atom. However, given the fact that the samples are grown in an oxygen deficient environment, which favors the formation of oxygen vacancies, we use the chemical potentials for "O-deficient" conditions and find that the cluster of Cu−O vacancies as shown in **Figure 3a** with a formation energy of 1.13 eV per Cu atom to be the most favorable defects to balance the stoichiometry. The Cu vacancies at CuO_2 planes, on the other hand, have a formation energy of 1.40 eV per Cu atom under O-deficient conditions. Consequently, from the DFT calculations, we find that oxygen vacancies play a significant role in stabilizing the Cu−O vacancy clusters as without them the stoichiometry would have to be balanced by creating Cu vacancies in the CuO_2 planes, which would have an adverse effect on the superconductive properties.

We have listed the formation energy (under O-deficient conditions) of all the different defects that we have calculated in **Table S1.**

Electronic Structure. The effect of the defects on the electronic structure was studied within the approximations described above. In order to understand the effect of the stacking faults in the electronic structure, we compared the linear charge density of the $YBa_2Cu_4O_8$ (Y-248) a supercell with and without $2V_{Cu}$+$3V_O$. The linear charge density is obtained by numerically integrating the charge density in the planes parallel to the CuO_2 plains where superconductivity takes place. The electronic densities for these two cases are shown in **Fig. S6.** Although the combinations of Cu and O vacancies that appear in the double chain do affect the charge density around the double chain that contains the vacancy, the charge density around the

superconducting planes is very similar for both cases. The main difference is actually due to the slightly smaller c parameter when the vacancies are considered. Also, note that the magnetic density in Fig. 3b of the main text appears only when the vacancies within the double chain are considered (it is zero for Y248), despite the overall similar electronic density.

S3. XMCD analysis

XMCD sum rules. XAS&XMCD measurements as a function of the temperature (at 6T) and as a function of the magnetic field (at 1.6 K) were performed in normal beam incidence (θ=0°). The sum rules[4,5] were applied to the background subtracted XAS spectra to evaluate the Cu orbital and effective spin moment:

$$m_L(\theta) = -2n_h \frac{q(\theta)}{r(\theta)}\mu_B \,, \qquad [1]$$

$$m_S^{eff}(\theta) = -3n_h \frac{\left[3p(\theta) - 2q(\theta)\right]}{r(\theta)}\mu_B \,, \qquad [2]$$

where n_h is the number of holes in the 3d shell, which we assumed to be n_h=1 for Cu^{2+} sites ($3d^9$). The orbital moment is defined by the expectation value of the ***L*** angular momentum operator: $m_L(\theta) = -\langle L\rangle(\theta)\mu_B/\hbar$, whereas the effective spin moment, $m_S^{eff}(\theta) = m_S - 7m_T(\theta)$, relates the isotropic spin moment $m_S = -2\langle S\rangle\mu_B/\hbar$ and the angle dependent $m_T(\theta) = \langle T\rangle(\theta)\mu_B/\hbar$, with ***S*** is the spin operator and ***T*** is intra-atomic spin dipole operator.

The integrals $p(\theta)$, $q(\theta)$ and $r(\theta)$ are defined as:

$$q(\theta) = \int_{L_{2,3}} I^-(E,\theta) - I^+(E,\theta)dE \,, \qquad [3]$$

$$p(\theta) = \int_{L_3} I^-(E,\theta) - I^+(E,\theta)dE \,, \qquad [4]$$

$$r(\theta) = \int_{L_{2,3}} I^-(E,\theta) + I^+(E,\theta) + I^0(E)dE \,, \qquad [5]$$

$I^0(E)$ being the isotropic absorption intensity measured with the photon linear polarization vector perpendicular to the direction of the incident light, approximated as $I^0(E) = \left(I^-(E,\theta) + I^+(E,\theta)\right)/2$.

Finally, the orbital to effective-spin ratio is determined as:

$$m_L / m_S^{eff} = \frac{2q(\theta)}{9p(\theta) - 6q(\theta)}, \qquad [7]$$

and thus is independent of the number of holes n_h and from the factor $r(\theta)$.

Figure S7a shows the effective spin and orbital moment field dependence, $m_S^{eff}(B)$ and $m_L(B)$, obtained from XMCD(*B*) measurements at 1.6 K, for the two studied samples. Note that the effective spin moment increases almost linearly with the field but tends towards saturation for very high fields (>4T). The orbital-to-effective spin moment ratio, at high fields, where the error is smaller, is close to $m_L / m_S^{eff}(B) \approx 0.22$, the value predicted from atomic model calculations for Cu^{2+} with a x^2-y^2 ground state[6].

Effective spin and spin moments. As extensively discussed in Ref.[6,7], for Cu^{2+} sites with high anisotropy, the effective spin moment $m_S^{eff}(\theta) = m_s - 7m_T(\theta)$ differs from the isotropic spin moment (m_s), since the angle dependent intra-atomic spin dipole moment (m_T) is non-negligible[6,7]. The spin-dipole moment expresses the inhomogeneous spatial distribution of the spin density over the atomic unit cell, due to the anisotropic charge distribution arising from strongly directional bonds or crystal field[8].

We adopted a methodology[9], previously applied to highly-anisotropic atoms on surfaces[10,11], to experimentally determine the isotropic moment (m_s) from XAS&XMCD measurements performed in grazing incidence, under the so-called "magic-angle" (θ^*=54.7^o). The measured XMCD with *B* parallel to the beam direction and incidence angle θ gives the projection of the Cu magnetic moment along the direction of the applied field. The anisotropic effective spin moment determined in grazing incidence can be written as:

$$m_S^{eff}(\theta^*) = m_s - 7m_T^z\left[\cos^2\theta - (1/2)\sin^2\theta\right]$$

where m_T^z is the z-component of the dipolar term parallel to the Cu fourfold axis C_4, and it has been assumed that in this symmetry $m_T^z + 2m_T^{xy} = 0$. At the magic angle (θ^*=54.7^o), such that $\cos^2\theta = (1/2)\sin^2\theta$, the dipolar term cancels, so $m_S^{eff}(\theta^* = 54.7^o)$ provides directly m_S [8].

For the evaluation of $m_S^{eff}(\theta^* = 54.7^o)$ in grazing incidence, we used Eq. [2] with the angular-corrected integral:

$$r'(\theta) = \int_{L_{2,3}} \frac{I^-(E,\theta)+I^+(E,\theta)}{f(\theta)} dE \,. \qquad [6]$$

where $f(\theta) = (\cos^2\theta + 1)/2$. As pointed out by Stepanow[7], this correction needs to be done for very anisotropic atoms to account for the angular dependence of the absorption intensity due to the spatial distribution of the 3d-orbitals. XAS&XMCD measurements in grazing incidence at the "magic angle" (θ^*=54.7°) performed on the Y123 standard sample at 6T and 1.6K (**Figure S7b**) yielded an isotropic moment value of $m_S = 0.064 \pm 0.005\mu_B$.

Total magnetic moment of the cluster. The total moment of the cluster (μ_c) is the moment (in μ_B units) of the ensemble of 4 magnetic Cu atoms surrounding the complex defect $2V_{Cu}$+$3V_O$ (Fig. 3).

Theoretically, it is calculated from DFT. The considered DFT simulation cell contains 16 Cu atoms, so when a $2V_{Cu}$+$3V_O$ appears, 14 Cu atoms are left. Only the 4 Cu atoms neighboring the defect carry a spin-only moment of ~0.325 μ_B each. The orbital magnetic moment (not included in the DFT simulations) adds an additional 20% contribution, according to our experiments. So the cluster moment expected from DFT is:

$$\mu_c^{DFT} = 4\times(0.325\mu_B + 20\%0.325\mu_B) \approx 1.56\mu_B$$

Experimentally, it is determined by fitting m_{TOT}(B) data to Eq. [1], within the model of superparamagnetic clusters including ferromagnetically ordered moments; the curvature of Langevin's function is directly related to the cluster moment, and gives μ_c=1.18±0.20μ_B for every sample, at every spot measured. This value is in fair agreement with theory, within all approximations made.

We estimate from DFT the saturation moment (per average Cu) that would be expected if all Cu atoms would belong to cells of the type described in Figure 3:

$$m_{TOT}^{sat,DFT} = \frac{\text{Moment of magnetic Cu around defect}}{\text{All Cu atoms in defected-cell}} = \frac{4\times(0.325\mu_B + 20\%0.325\mu_B)}{14} = \frac{1.56\mu_B}{14} \approx 0.11\mu_B / Cu$$

Calling f the fraction of Cu atoms in the sample that belong to defect containing-cells, the expected saturation moment/Cu to be measured by XMCD would be:

$$m_{TOT}^{sat,XMCD} = f.m_{TOT}^{sat,DFT} = f\cdot 0.11\mu_B$$

From the fit of experimental data to Langevin's function, the determined saturation moment/Cu ranges from 0.06 – 0.15 μ_B, with differences observed from sample-to sample and within one sample. It is concluded that *f*~1, i.e., a major fraction of the sample sensed by TEY contains faulted cells. Considering all the approximations made, this figure just indicates that a major fraction of sample-surface has the defected-structure described by DFT, giving rise to magnetism.

Table S1. Formation energy of different defects calculated under O-deficient conditions. The site of the defects can be identified using the following key: The total number of vacancies (*n*) in the defect is denoted by *n*V and is mentioned at the beginning. This is followed by the type of vacancies where O and Cu represent oxygen and copper vacancy, respectively. The location of the vacancy follows the type of vacancy and is denoted by either Ba, sch, ch1, ch2 or pl, which represent BaO plane, single CuO chain, chain1 of the double CuO chain, chain2 of the double CuO chain, and CuO_2 plane, respectively. For Cu vacancies and Cu+O vacancy combinations the formation energies are given per Cu vacancy. Similarly, for single and multiple oxygen vacancies, the formation energies are expressed per O vacancy. The most favorable defect in each class has been highlighted with bold text.

Defect	Formation energy (eV)
**O vacancies**	
1V_OBa	1.13
**1V_Osch**	**−0.38**
1V_Och1	0.46
1V_Opl	0.75
2V_Osch_Osch	−0.42
2V_Och1_Och1	0.21
2V_Och1_Och2	0.61
3V_Och1_Och1_Och2	0.60
4V_Och1_Och1_Och2_Och2	0.76
**Cu vacancies**	
**V_Cupl**	**1.40**
V_Cuch1	2.11
2V_Cuch1_Cuch1	2.95

2V_Cuch1_Cuch2	**2.47**
<u>**Cu+O vacancies**</u>	
4V_Cuch1_Cuch2_Och1_Och1	1.51
4V_Cuch1_Cuch2_Och1_Och2	1.25
3V_Cuch1_Cuch2_OBa	2.03
3V_Cuch1_Cuch2_Opl	2.59
3V_Cuch1_Cuch2_Och1	1.86
4V_Cuch1_Cuch2_Och1_Opl	2.17
4V_Cuch1_Cuch2_Opl_Opl	2.93
2V_Cuch1_Och1	2.33
2V_Cuch1_Och2 (near)[a]	1.56
2V_Cuch1_Och2 (far)[b]	2.10
2V_Cupl_Opl	1.49
**5V_ Cuch1_Cuch2_Och1_Och1_Och2**	**1.13**
6V_Cuch1_Cuch2_Och1_Och1_Och2_Och2	1.69

[a.]The Cu vacancy and O vacancy are nearest neighbor and form a defect complex.

[b.]The Cu vacancy and O vacancy are not nearest neighbors

Supplementary Figures

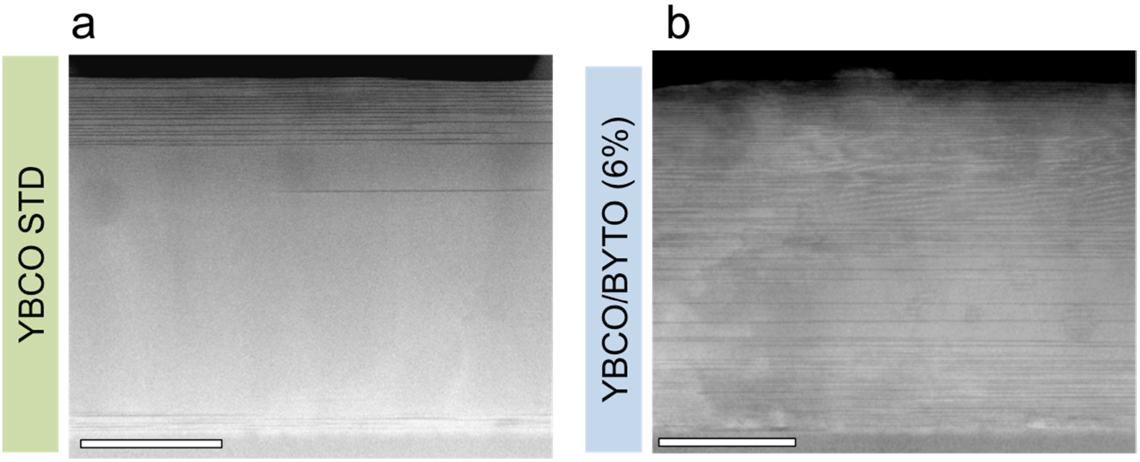

Figure S1. STEM images of the microstructure of a standard and a nanocomposite films**. a)** and b), comparison of the microstructure between a pristine YBCO film and YBCO-6% BYTO nanocomposite films, respectively. Y124 intergrowths appear as dark stripes in high resolution Z-contrast images. Scale bar 50 nm.

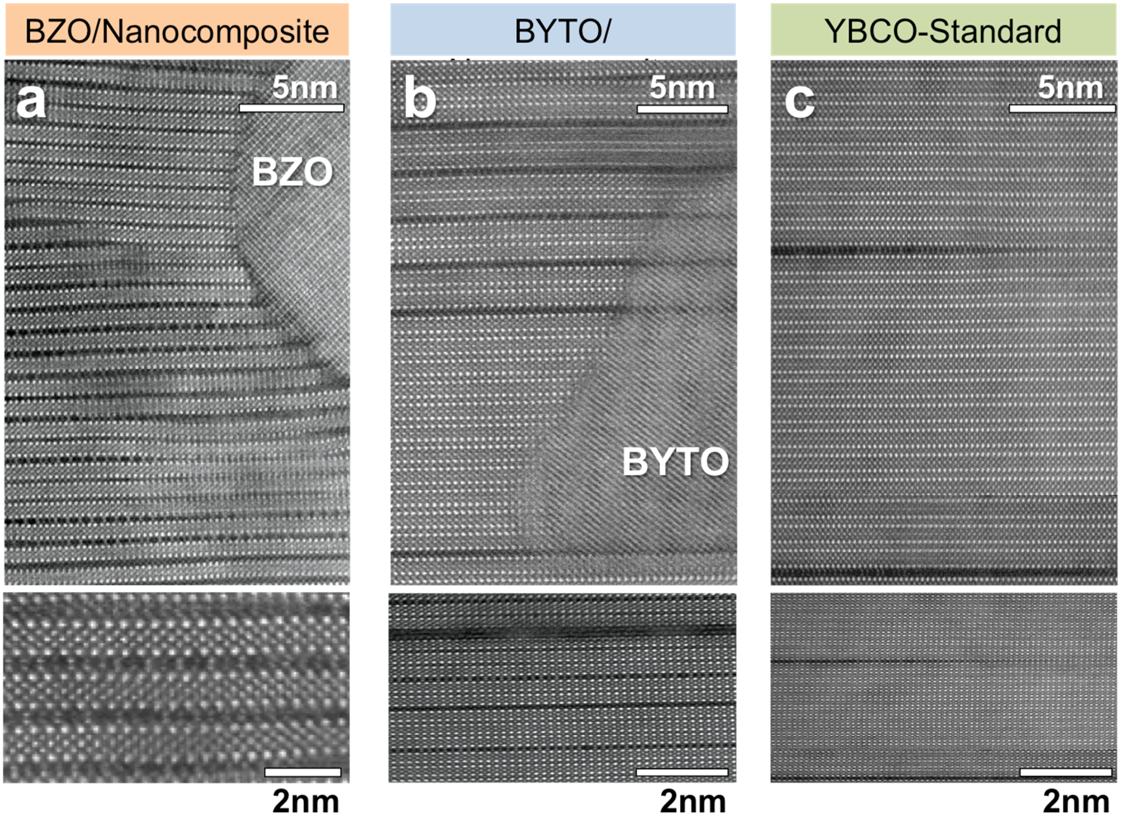

Figure S2. High resolution STEM images of the local microstructure in Y123 nanocomposites and in a standard film. a), b) and c) (upper panel) show three high resolution HAADF images of a Y123-BZO nanocomposite film, a Y123-BYTO nanocomposite film and a standard film, respectively. Lower panels are higher magnification images of these samples, showing the presence of Cu vacancies in each of them.

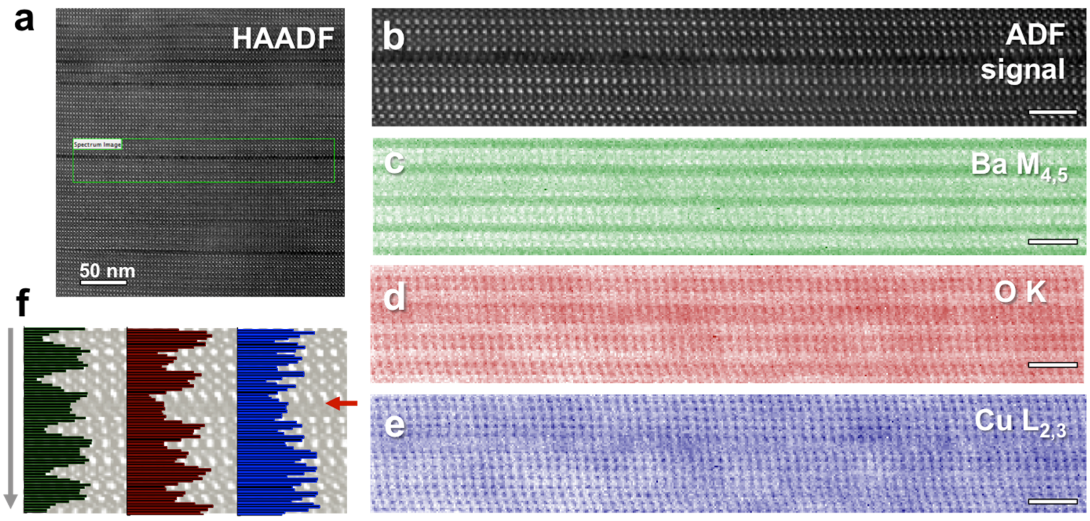

Figure S3. STEM-EEL spectrum image. a), High resolution Z-contrast image of a Y123 nanocomposite thin film. The square marks the area where the spectrum image was acquired. b), simultaneous ADF signal. **c-e**, 2D EELS maps corresponding to the Ba $M_{4,5}$ edge c), O K edge d) and Cu $L_{2,3}$ edges e). The integrated intensity of each row of pixels in the raw Ba (in green), O (in red) and Cu (in blue) maps is shown. The red arrow points to the location of the double Cu-O chain. Scale bars, 4 nm.

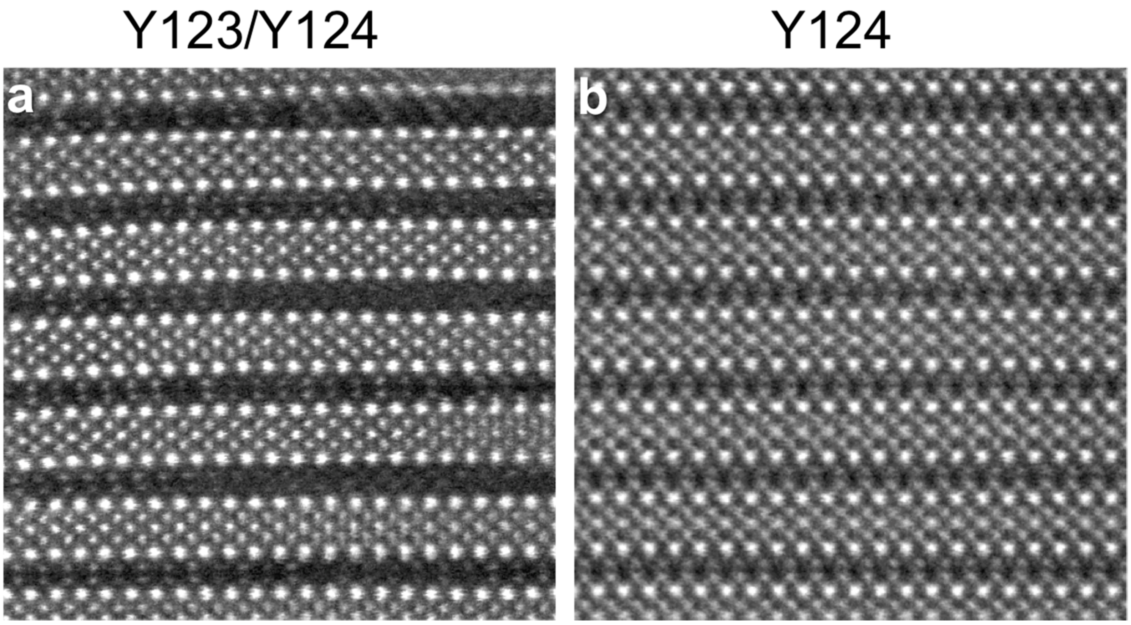

Figure S4. High resolution STEM images of the local microstructure of Y123 nanocomposites and of a Y124 film. a) and b), HAADF images of the Y124 phase from two different samples, a Y123 nanocomposite film and a Y124 film, respectively.

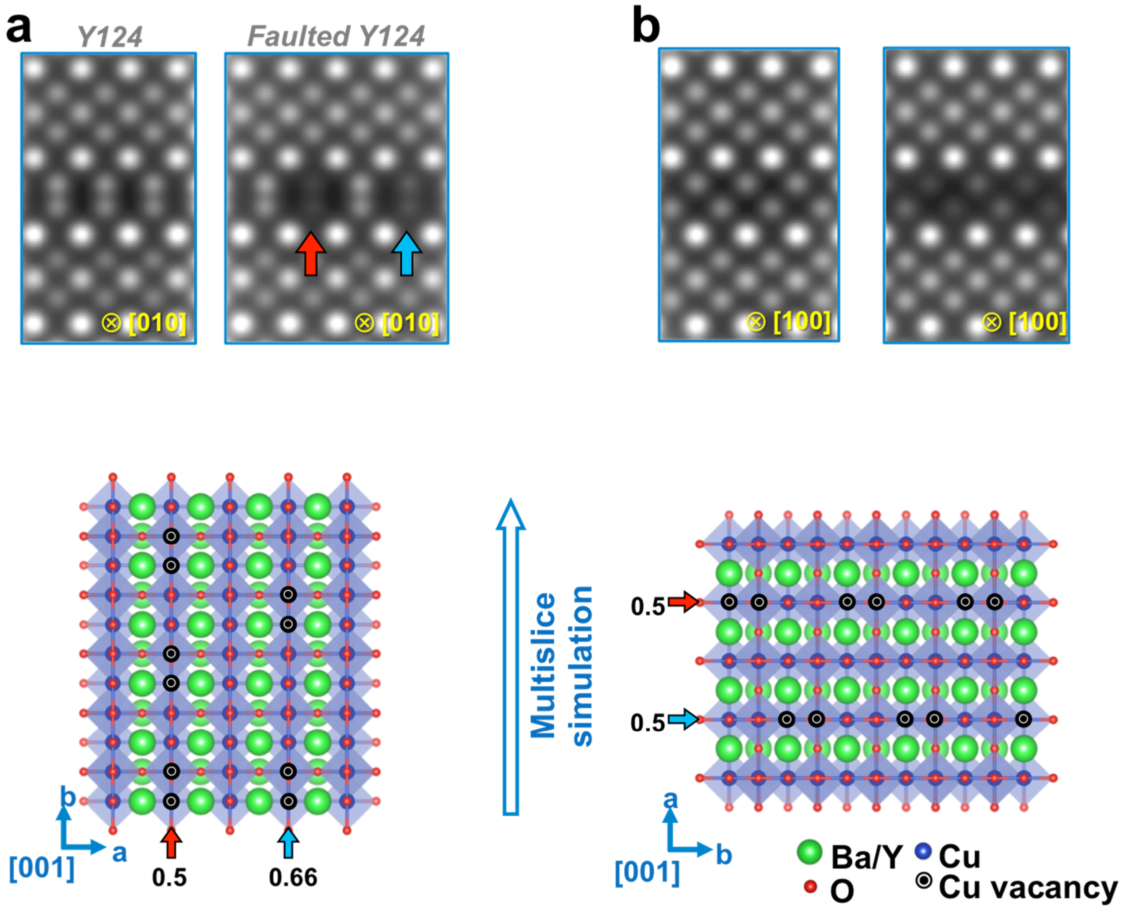

Figure S5. Simulated HAADF images of the faulted and fully stoichiometric Y124 phases. a) and b), Y124 intergrowth images viewed along the [010] and [100] zone axis, respectively. The simulated image of faulted Y124 in a) is performed considering a Cu occupancy of 0.5 and 0.66 in the second (red arrow) and fourth (blue arrow) Cu-pair columns, respectively. The simulated image of faulted Y124 in b) is performed considering a Cu occupancy of 0.5 in two non-consecutive (010) Y124 planes. For both cases, an approximately 3.5nm thick supercell was created for the simulations. Lower panels show a [001] view of the faulted Y124 supercell used for HAADF simulations. Cu vacancies are represented by black dots. Notice that in order to achieve a Cu occupancy of 0.5 and 0.66, one Cu-pair of every two pairs (red arrow) and every three pairs (blue arrow) are removed.

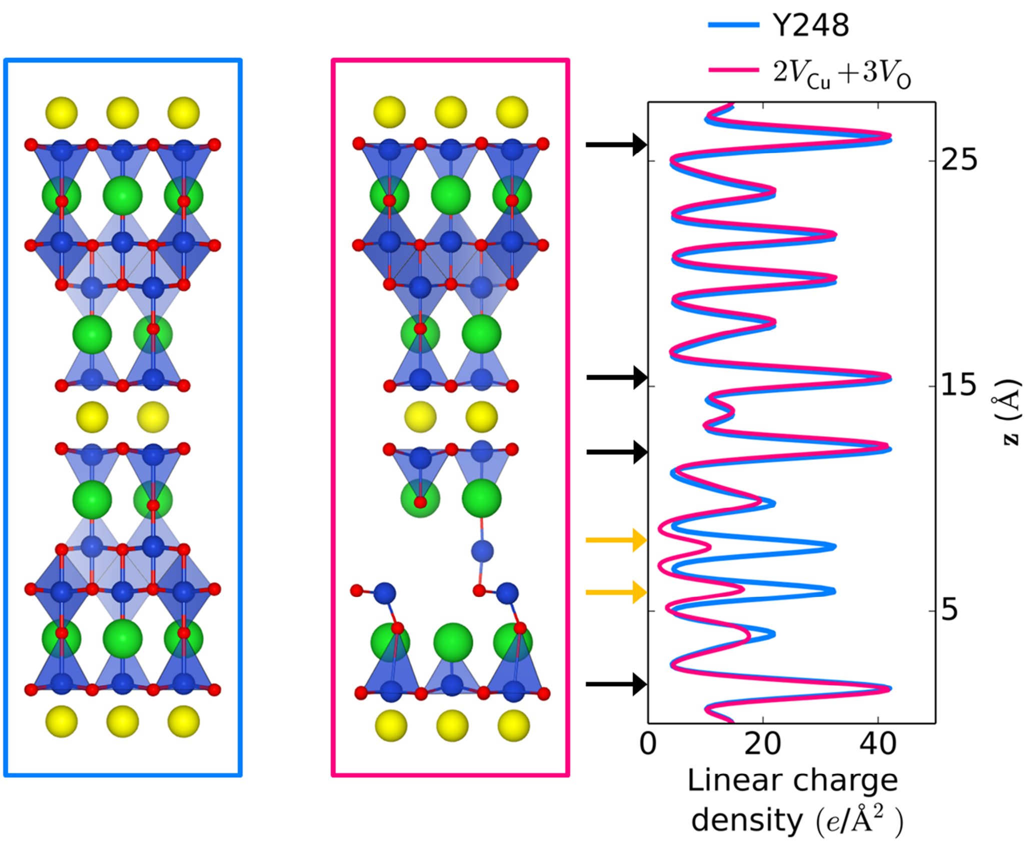

Figure S6. Charge density around the $2V_{Cu}$+$3V_O$ defect. Left and medium panel show the atomic structure around the double chain in the stoichiometric Y248 (left, blue frame) and the Y248 structure with the decorated Cu di-vacancies (middle, pink frame). Right panel shows the linear charge density for both cases in the direction perpendicular to the CuO_2 planes. Superconducting CuO_2 planes are indicated by black arrows, CuO_{1-x} chains by orange arrows. Note that there are significant differences in the charge density around the double chain due to the vacancies, but in the CuO_2 planes the charge density is very similar.

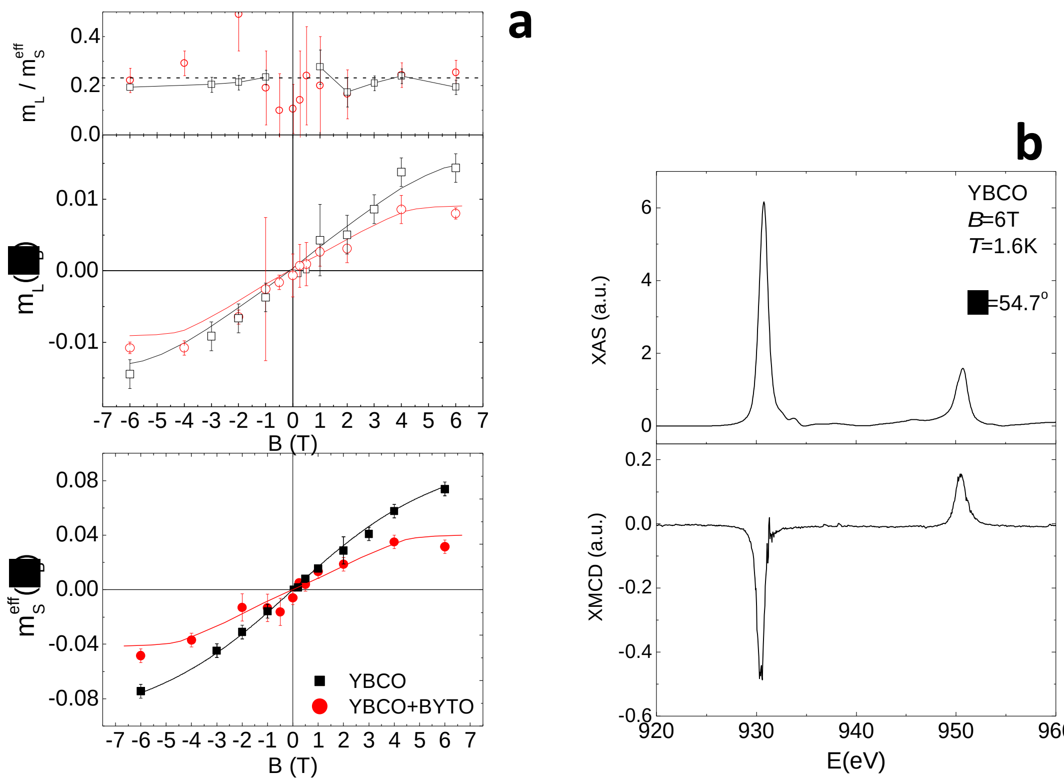

Figure S7. a) Magnetic field dependence of the (bottom) effective spin moment, (middle) orbital moment and (top) orbital-to-effective spin moment ratio for the standard YBCO sample at 1.6K, in normal incidence (θ^{*}=54.7°)**;** b) Cu $L_{2,3}$ edge background-subtracted XAS and XMCD spectra measured on standard YBCO standard at 6 T, 1.6 K in grazing incidence, at the magic angle (θ^{*}=54.7°).

References

[1] V. Grillo, E. Rotunno, *Ultramicroscopy* **2013**, *125*, 97.

[2] E. J. Kirkland, *Advanced Computing in Electron Microscopy*, Springer, New York, **1998**.

[3] S. Zhang, J. Northrup, *Phys. Rev. Lett.* **1991**, *67*, 2339.

[4] B. T. T. Thole, P. Carra, F. Sette, G. Van der Laan, *Phys. Rev. Lett.* **1992**, *68*, 1943.

[5] P. Carra, B. T. Thole, M. Altarelli, X. Wang, *Phys. Rev. Lett.* **1993**, *70*, 694.

[6] C. Piamonteze, P. Miedema, F. M. F. De Groot, *Phys. Rev. B - Condens. Matter Mater. Phys.* **2009**, *80*, 1.

[7] S. Stepanow, A. Mugarza, G. Ceballos, P. Moras, J. C. Cezar, C. Carbone, P. Gambardella, *Phys. Rev. B - Condens. Matter Mater. Phys.* **2010**, *82*, DOI 10.1103/PhysRevB.82.014405.

[8] J. Stöhr, H. König, *Phys. Rev. Lett.* **1995**, *75*, 3748.

[9] G. van der Laan, *Phys. Rev. B* **1998**, *57*, 5250.

[10] J. Bartolomé, F. Bartolomé, L. M. García, G. Filoti, T. Gredig, C. N. Colesniuc, I. K. Schuller, J. C. Cezar, *Phys. Rev. B - Condens. Matter Mater. Phys.* **2010**, *81*, DOI 10.1103/PhysRevB.81.195405.

[11] P. Gargiani, G. Rossi, R. Biagi, V. Corradini, M. Pedio, S. Fortuna, A. Calzolari, S. Fabris, J. C. Cezar, N. B. Brookes, M. G. Betti, *Phys. Rev. B - Condens. Matter Mater. Phys.* **2013**, *87*, DOI 10.1103/PhysRevB.87.165407.
